# Preventive Effects of Fermented Brown Rice and Rice Bran against Prostate Carcinogenesis in TRAP Rats

**DOI:** 10.3390/nu8070421

**Published:** 2016-07-11

**Authors:** Toshiya Kuno, Aya Nagano, Yukiko Mori, Hiroyuki Kato, Yuko Nagayasu, Aya Naiki-Ito, Shugo Suzuki, Hideki Mori, Satoru Takahashi

**Affiliations:** 1 Department of Experimental Pathology and Tumor Biology, Nagoya City University Graduate School of Medical Sciences, 1-Kawasumi, Mizuho-cho, Mizuho-ku, Nagoya 467-8601, Japan; me_me_sheep3@yahoo.co.jp (A.N.); ymori345@gmail.com (Y.M.); h.kato@med.nagoya-cu.ac.jp (H.K.); naga-p@dk.pdx.ne.jp (Y.N.); ayaito@med.nagoya-cu.ac.jp (A.N.-I.); shugo@med.nagoya-cu.ac.jp (S.S.); sattak@med.nagoya-cu.ac.jp (S.T.); 2 A former president of Gifu University, 1-1 Yanagido, Gifu 501-1194, Japan; hidmori@gifu-u-ac.jp

**Keywords:** fermented brown rice and rice bran with *Aspergillus oryzae* (FBRA), prostate cancer, transgenic rat

## Abstract

Fermented brown rice and rice bran with *Aspergillus oryzae* (FBRA) is considered to have the potential to prevent chemically-induced carcinogenesis in multiple organs of rodents. In the present study, we evaluated the possible chemopreventive effects of FBRA against prostate tumorigenesis. Six-week-old male rats of the transgenic rat for adenocarcinoma of prostate (TRAP) strain were fed diets containing 5% or 10% FBRA for 15 weeks. Animals were sacrificed at 21 weeks of age, and the ventral and lateral prostate were removed for histopathological evaluation and immunoblot analyses. FBRA decreased the incidence of adenocarcinoma in the lateral prostate and suppressed the progression of prostate carcinogenesis. Treatment with FBRA induced apoptosis and inhibited cell proliferation in histologically high-grade prostatic intraepithelial neoplasias. Phospho-AMP-activated kinase α (Thr172) was up-regulated in the prostate of rats fed the diet supplemented with FBRA. These results indicate that FBRA controls tumor growth by activating pathways responsive to energy deprivation and suggest that FBRA has translational potential for the prevention of human prostate cancer.

## 1. Introduction

Prostate cancer is the most frequently diagnosed malignancy and the second most common cause of cancer-related death in elderly men in the United States [1]. Although its incidence varies across the globe, its incidence and associated mortality are strikingly lower in the Asia–Pacific region when compared with Western countries [2]. According to autopsy-based prevalence data, the incidence of latent prostate cancer in Asia is still low despite a rapid increase in the incidence of clinical malignancy [3]. It has been suggested that one of the etiologic factors for the high incidence of prostate cancer in Western countries is a high dietary intake of highly processed foods [4].

Brown rice is a staple dietary constituent in Asia, whereas rice consumed in the Western world is generally white rice obtained from brown rice by removal of the bran. Rice seeds and rice bran contain fiber and several kinds of useful micronutrients, such as phenolic acids including ferulic acid, phytic acid (inositol hexaphosphate), tocopherols, and tocotrienols [5]. A large number of cellular and preclinical studies have supported that micronutrients in rice bran protect against the occurrence of cancer [6,7]. Fermented brown rice and rice bran with *Aspergillus oryzae* (FBRA) is a food manufactured by fermenting a mixture of brown rice and rice bran with *A. oryzae* to improve its digestibility. *A. oryzae* is known to contain several types of enzymes that metabolize carbohydrates and proteins, thereby producing a variety of functional substances during fermentation. To date, FBRA has been shown to have chemopreventive effects against carcinogenesis in the esophagus, stomach, colon, liver, lung, pancreas and bladder of rodents [8,9,10,11,12,13,14]. Since FBRA has no systemic toxicity and does not contain artificial chemicals, it is attractive as a functional food, and examination to determine whether it has a chemopreventive effect against prostate carcinogenesis will be valuable.

The chemoprevention of prostate cancer would be of great benefit, especially since the clinical behavior of the disease is heterogeneous and large subgroup populations of the disease exist. In the present study, we aimed to elucidate the chemopreventive effect of FBRA exposure against prostate cancer growth using the transgenic rat for adenocarcinoma of prostate (TRAP) model. In TRAP male rats, the hormonally regulated rat probasin promotor specifically drives the expression of SV40 T antigen (Tag) in the prostatic epithelium, causing the spontaneous induction of neoplastic transformation in almost all acini in the prostatic lobes [15]. SV40 Tag abrogates the functions of the tumor suppressors p53 and RB, leading to the development of spontaneous progressive prostatic intraepithelial neoplasia (PIN), which results in non-invasive adenocarcinomas by 15 weeks of age [16]. This tumorigenesis process closely simulates the progressive steps of human prostate carcinoma. Therefore, a short-term experimental study using the TRAP model was considered to be a good tool for evaluating whether FBRA has a chemopreventive action against prostate carcinogenesis. We previously reported that phytochemicals such as ellagic acid, apocynin, nobiletin, and auraptene had suppressive effects on prostate carcinogenesis in the TRAP model [17,18,19].

In this study, the potential chemopreventive effect of FBRA was examined by focusing on the early stages of prostate carcinogenesis. The results provided evidence that FBRA can suppress prostate carcinogenesis with induction of apoptosis and inhibition of proliferation through activating pathways responsive to energy deprivation. Consequently, we hope that the present findings regarding the effect of FBRA in the TRAP model could have clinical significance.

## 2. Materials and Methods

### 2.1. Animals

Male heterozygous TRAP rats established in our laboratory with a Sprague–Dawley genetic background were used in the present study. All animals were housed in plastic cages on wood-chip bedding in an air-conditioned specific pathogen-free animal room at 22 ± 2 °C and 55% ± 5% humidity with a 12 h light/dark cycle. The animals were fed a basal diet (Oriental MF, Oriental Yeast, Tokyo, Japan) and provided with water ad libitum. All animal experiments were performed under protocols approved by the Institutional Animal Care and Use Committee of Nagoya City University School of Medical Sciences (No. H26M-03; approved on 2 April 2014).

Six-week-old TRAP male rats (*n* = 36) were divided into 3 groups of 12 rats each. FBRA was obtained from Genmai Koso Co. Ltd., (Sapporo, Japan) and freshly prepared for administration to the treatment groups. The animals were fed a diet containing 0%, 5%, or 10% FBRA for 15 weeks, and body weight and food consumption were estimated weekly. At experimental week 15, under deep isoflurane anesthesia, blood was collected from 9:00 to 11:00 h to measure testosterone and estradiol levels using chemiluminescence immunoassays obtained from a commercial laboratory (The Tohkai Cytopathology Institute, Gifu, Japan). The urogenital complex of each rat was removed as a whole together with the seminal vesicles, then the ventral prostate (VP) was weighed. A part of the prostate glands was immediately frozen in liquid nitrogen and stored at −80 °C until processed, and the remaining tissue was fixed in formalin. The liver and kidneys were also removed, weighed, and fixed. The tissues were routinely processed into paraffin-embedded sections and stained with hematoxylin and eosin.

### 2.2. Assessment of Prostate Neoplastic Lesion Development

Neoplastic lesions in the prostate gland of TRAP rats were classified into 3 types: low-grade PIN (LG-PIN), high-grade PIN (HG-PIN), and adenocarcinoma, as previously described by Seeni et al. [20]. The incidence of neoplastic lesions was scored in tissue sections of the VP and lateral prostate (LP). The relative numbers of acini with the histological characteristics of LG-PIN, HG-PIN, and adenocarcinoma were quantified by counting the acini in each prostatic lobe.

### 2.3. Immunohistochemistry

Deparaffinized sections were incubated with diluted antibodies for androgen receptor (Santa Cruz Biotechnology, Dallas, TX, USA, catalog #sc-816, 1:500 dilution), Ki-67 (Abcam, Cambridge, UK, #ab16667, 1:100), and anti-phospho-AMP activated kinase (AMPK) α antibody (Thr172, Cell Signaling Technology (CST), Danvers, MA, USA, #2535, 1:100). Apoptotic cells were detected by terminal deoxy nucleotidyl transferase-mediated dUTP nick end labeling (TUNEL) assay using an in situ Apoptosis Detection Kit from Takara Bio Inc. (Otsu, Japan). The labeling indices of Ki-67 and TUNEL were determined by counting at least 1000 HG-PIN cells under a microscope light microscopy at high magnification. Phospho-AMPKα-positive cells were graded by immunoreactivity (intensity of brown staining) as 0 (no staining), +1 (very weak), +2 (weak), +3 (moderate), and +4 (strong), as previously described by Raina K, et al. [21].

### 2.4. Western Blot Analysis

The harvested frozen tissues were homogenized in radioimmunoprecipitation assay buffer (150 mM NaCl, 0.5% sodium deoxycholate, 1% NP-40, 0.1% SDS, and 50 mM Tris-HCl) containing a protease inhibitor (1 mM phenylmethylsulfonyl fluoride). Total cellular proteins were quantified by the Bradford procedure and equal amounts of proteins were mixed with Laemmli sample buffer (Bio-Rad Laboratories, Inc., Hercules, CA, USA) and fractionated by gel electrophoresis in 6% or 12% polyacrylamide resolving gels containing 0.1% SDS. Proteins were transferred onto Hybond^®^ ECL™ nitrocellulose membranes (Amersham Biosciences, Little Chalfont, UK), which were subsequently incubated overnight with primary antibodies against phospho-AMPKα (Thr172, CST, #2535, 1:1000), total AMPKα (CST, #5831, 1:1000), fatty acid synthase (CST, #3180, 1:1000), cyclin D1 (Santa Cruz Biotechnology, #sc-753, 1:200), caspase 3 (CST, #9662, 1:1000), caspase 7 (CST, #9492, 1:1000), Erk1/2 (CST, #9102, 1:1000), phospho-Erk1/2 (Thr202/Tyr182, CST, #9101, 1:1000), p38 MAPK (CST, #9212, 1:1000), and phospho-p38 MAPK (Thr180/Tyr182, CST, #5831, 1:1000). Equal protein loading was ascertained by western blotting with an anti-β-actin antibody (Sigma-Aldrich, St. Louis, MO, USA, #A5316, 1:5000). The signal was detected using an ImageQuant™ LAS-4000 mini biomolecular imager (GE Healthcare, Little Chalfont, UK). The intensity of each band was measured using ImageJ 1.48v (National Institutes of Health, Bethesda, MD, USA). Each western blot analysis was performed three times to confirm reproducibility.

### 2.5. Statistical Analysis

The data were analyzed using 1-way analysis of variance followed by Dunnett’s test. The data were expressed as mean ± standard deviation and differences between groups were considered to be significant at *p* < 0.05. All statistical analyses were performed using GraphPad Prism software (version 6; GraphPad Software, Inc., La Jolla, CA, USA).

## 3. Results

### 3.1. FBRA Suppressed Progression of Prostate Carcinogenesis without Toxicity

**Figure 1:**
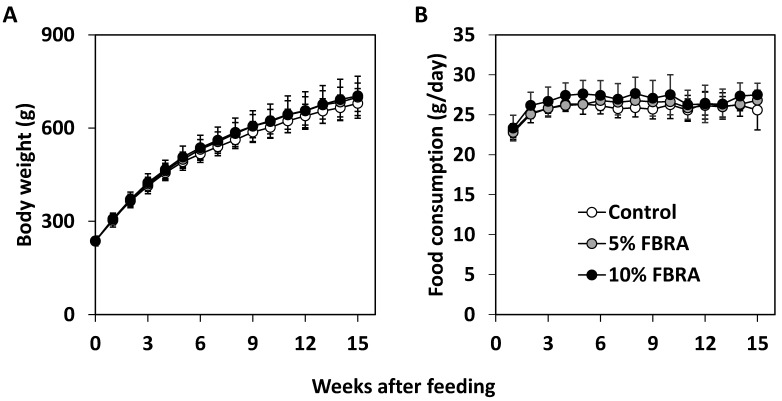
(**A**) Effect of exposure to fermented rice and rice bran with Aspergillus oryzae (FBRA) on the body weight of transgenic rat for adenocarcinoma of prostate (TRAP) rats. Body weights of rats are represented as the average of each group and plotted as a function of time (weeks) for each group; (**B**) Effect of FBRA on daily food consumption by TRAP rats. Food consumption by each rat (grams per day) was recorded weekly throughout the experimental period in each group.

Administration of FBRA did not affect body weight or the relative weights of the liver and VP (Figure 1 and Table 1). Although the mean weight of the kidneys in the 5% FBRA treated group was greater than that in the control group, it was not different between the 10% FBRA treated group and the control group. There were no histopathological changes to indicate toxicity of FBRA in the liver and kidneys.

**Figure 2:**
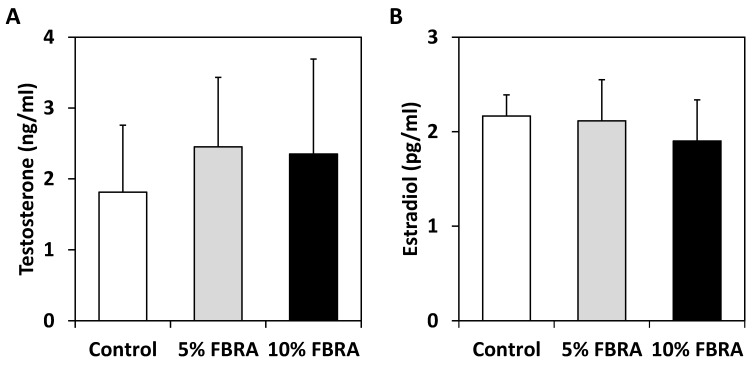
Levels of serum testosterone (**A**) and estradiol (**B**) in TRAP treated with FBRA.

Furthermore, no significant differences in serum testosterone and estrogen levels among groups were observed (Figure 2).

**Figure 3:**
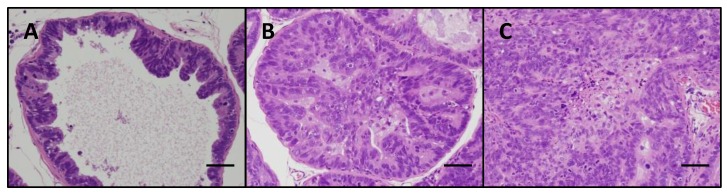
Representative histological findings for (**A**) low-grade prostatic intraepithelial neoplasia; (**B**) high-grade prostatic intraepithelial neoplasia; and (**C**) adenocarcinoma. Scale bars, 50 μm.

As shown in Figure 3, TRAP rats showed a sequential development of prostatic proliferative lesions that were histopathologically diagnosed as low-grade prostatic intraepithelial neoplasia (LG-PIN), high-grade (HG)-PIN, or adenocarcinoma.

**Figure 4:**
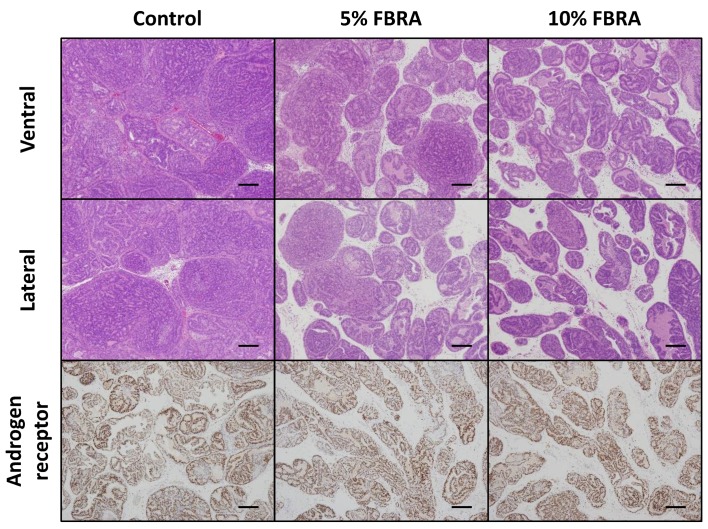
Effects of FBRA on prostatic lesions. Representative histopathological findings for lesions in the ventral prostate (VP) and lateral prostate (LP) of the control group and the 5% and 10% FBRA groups. The lower panels show immunohistochemical staining for androgen receptor. Scale bars, 200 μm.

Representative histopathological findings of VP and LP in each group are shown in Figure 4. There was a response to FBRA as demonstrated by the reduction in the number of prostate neoplastic lesions. Immunohistochemical analysis showed that there were no significant changes in androgen receptor expression at the protein level as a result of FBRA treatment.

Prostatic proliferative lesions and adenocarcinomas developed in all groups. The numbers of LG-PIN, HG-PIN, and adenocarcinoma lesions in VP and LP were scored by microscopy and presented as the percentage of total lesions in each prostate as summarized in Table 2.

The percentage of LG-PIN in the VP of the group fed 10% FBRA was significantly higher than that of the control group (12.9% ± 3.9% vs. 8.6% ± 3.5%, *p* < 0.01). Conversely, the percentage of adenocarcinoma in the VP of the group fed 10% FBRA was lower than that of the control group (12.1% ± 7.1% vs. 15.7% ± 9.1%). The percentage of adenocarcinoma in the LP of the group fed 10% FBRA was significantly lower than that of the control group (4.4% ± 5.3% vs. 9.4% ± 6.1%, *p* < 0.05). The total incidence of carcinoma in the LP of the group fed 10% FBRA (75%) was also lower than that of the control group, but this difference did not reach statistical significance. These data from quantitative evaluations of the proportion of neoplastic lesions in the prostate gland suggested that the transition from PIN to adenocarcinoma was significantly suppressed by the dietary consumption of FBRA.

### 3.2. Effects of FBRA on Cell Proliferation and Apoptosis

**Figure 5:**
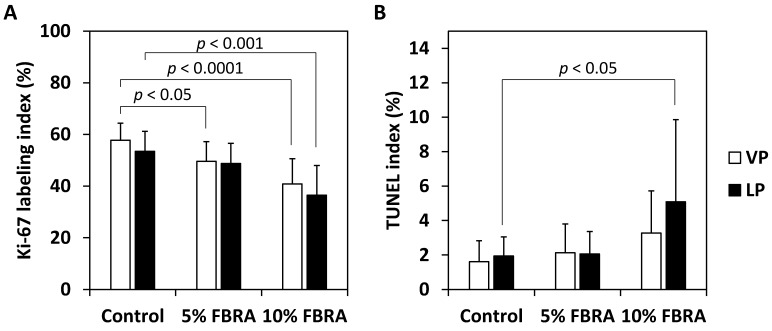
Labeling indices for (**A**) Ki-67- and (**B**) transferase-mediated dUTP nick end labeling (TUNEL)-positive cells in the VP and LP of TRAP rats.

The Ki-67 index, which indicates cell proliferation, of HG-PIN in the VP and LP of the group fed 10% FBRA was significantly lower than that of the control group (Figure 5A) (*p* < 0.0001 and 0.001, respectively). The apoptotic index of HG-PIN as determined by TUNEL assay in the LP of the group fed 10% FBRA was significantly higher than that of the control group (Figure 5B) (*p* < 0.05).

### 3.3. Effect of FBRA on Activation of AMPK

**Figure 6:**
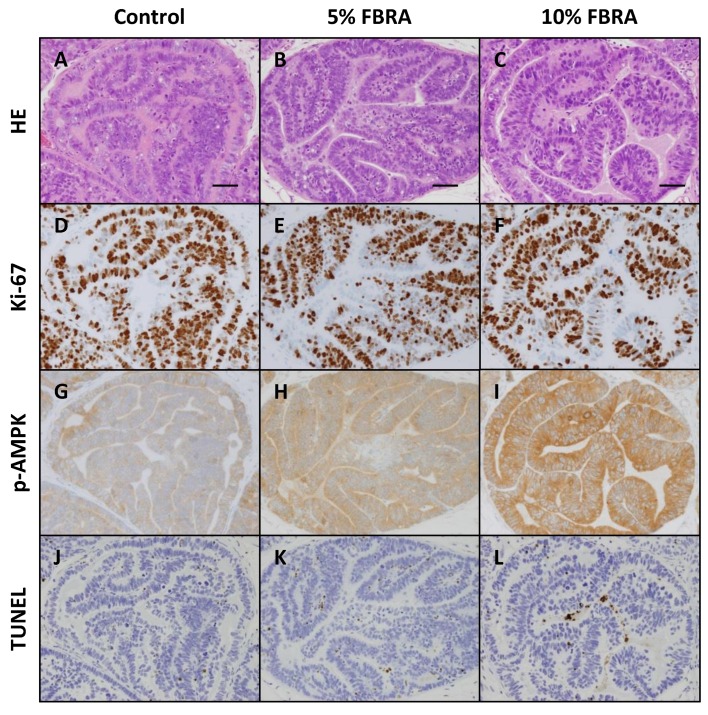
(**A**–**C**) Hematoxylin and eosin; (**D**–**F**) Ki-67 expression; (**G**–**I**) p-AMPK expression; and (**J**–**L**) TUNEL assay in high-grade prostatic intraepithelial neoplasia in the LP of TRAP rats. Immunoreactivity for p-AMPK was scored as (**G**) +1 (very weak); (**H**) +2 (weak); and (**I**) +3 (moderate). Scale bars, 50 μm.

**Figure 7:**
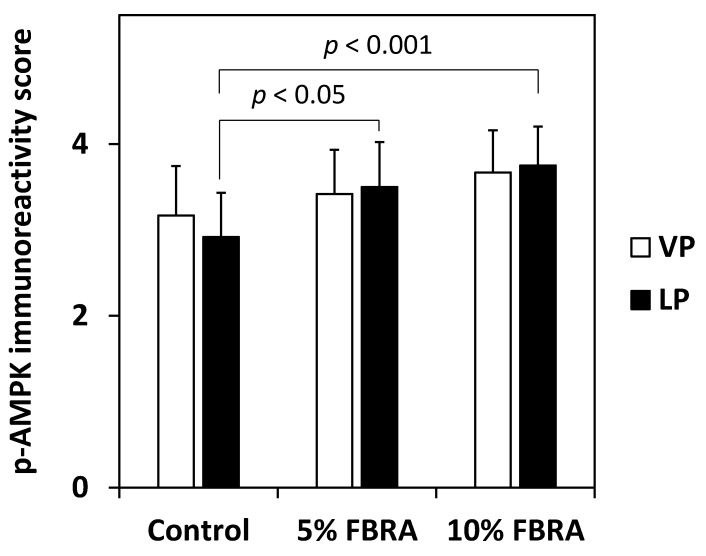
Dose-dependent effect of dietary FBRA on p-AMPK in HG-PIN in the LP of TRAP rats. Columns represent mean and standard deviation (error bars) in each group.

To further understand the mechanistic effect of FBRA on prostate carcinogenesis in TRAP rats, the hypothesis that FBRA activates AMPK was examined. The immunoreactivity scores for phospho-AMPK in HG-PIN in the lateral prostate of rats fed 5% and 10% FBRA were significantly increased compared with those of controls (*p* < 0.05 and 0.001, respectively) (Figure 6 and Figure 7).

**Figure 8:**
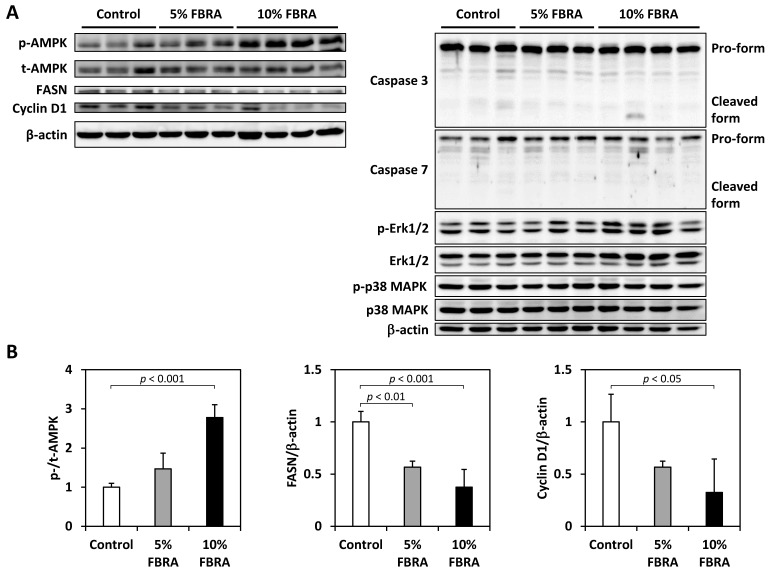
Immunoblot analysis in the LP of TRAP rats treated with FBRA. (**A**) Immunoblots of protein lysates (20μg) of LP were probed with antibodies against p-AMPK^Thr172^, total AMPK, fatty acid synthase (FASN), cyclin D1, caspase 3 and 7, p-Erk1/2, Erk1/2, p-p38 MAPK, p38 MAPK, and β-actin; (**B**) Bar charts show relative protein levels in the treatment and control groups. All data are expressed as a fold change relative to the control group.

Immunoblot analyses also demonstrated that p-AMPK expression was upregulated, while fatty acid synthase (FASN) and cyclin D1 expression were downregulated in the LP of rats treated with FBRA. Meanwhile, no changes in the expression of caspase 3 and 7, Erk1/2, or p38 MAPK were evident (Figure 8).

## 4. Discussion

The present study demonstrated a significant suppression of the induction of adenocarcinoma in TRAP rats by dietary exposure to 10% FBRA, suggesting that this agent has a potential chemopreventive effect on prostate carcinogenesis. Dietary consumption of FBRA by TRAP rats resulted in a shift in the pathological grade distribution of their prostatic lesions from adenocarcinomas towards precancerous lesions. It is suggested that FBRA blocks the transitions from LG-PIN to HG-PIN and from HG-PIN to adenocarcinoma.

FBRA has previously been proven to inhibit chemically-induced carcinogenesis in multiple organs of rodents. Various mechanisms by which chemopreventive agents exert their inhibitory effects on tumorigenesis have been considered. For example, cell proliferation is important in multistage carcinogenesis and becomes abnormal through multiple genetic alterations. In this study, FBRA inhibited cell proliferation and enhanced the induction of apoptosis in prostatic intraepithelial neoplasia. These results are in line with previous studies using cancer cell lines and animal models [8,9,10,11,12,13,14,22,23]. However, the possible mechanisms underlying the biological effects of FBRA have not yet been fully understood.

AMPK is a serine/threonine kinase that acts as an energy sensor and also links cell metabolism and oncogenesis. AMPK has recently emerged as a potential therapeutic target for cancer treatment, and its activators such as metformin have been shown to decrease the risk of prostate cancer-specific and all-cause mortality among diabetic men in a dose-dependent manner [24]. In this study, FBRA upregulated the expression of AMPK and downregulated the expression of FASN and cyclin D1 in prostate tumors. Zadra et al. reported that the AMPK activator MT63-78 decreased prostate tumor growth and inhibited FASN expression, and its antigrowth effects were mediated by the inhibition of lipogenesis in androgen-sensitive and castration-resistant models [25]. In addition, Fan et al. reported that (Z)3,4,5,4′-transtetramethoxystilbene, which is an analogue of resveratrol activated AMPK and induced caspase-independent apoptosis in gefitinib-resistant non-small-cell lung cancer cells. FBRA did not affect the cleavage of caspases 3 and 7, and it is possible that apoptosis in the prostate neoplastic lesions may be induced by FBRA in a similar caspase-independent manner [26]. The chemopreventive action of FBRA may be attributable to its ability to activate AMPK.

FBRA is a food prepared by the solid-state fermentation of brown rice and rice bran with *A. oryzae*. Rice bran contains a variety of bioactive components that are thought to have chemopreventive activity, including γ-oryzanol, ferulic acid, caffeic acid, phytic acid, coumaric acid, vitamin E isoforms (α- and γ-tocopherols and various tocotrienols), phytosterols, carotenoids (α- and β-carotene, lutein, and lycopene), cellulose, arabinoxylan, lignin, and β-glucan [6]. Previous studies have further implied that the process of fermenting rice bran with bacteria or fungi alters its bioactivity [27,28]. In our previous study, γ-tocopherol decreased the number of adenocarcinomas in the VP in TRAP rats through activating caspases and inactivating Erk1/2 [29]. Therefore, since the expression of caspases and Erk1/2 were not affected by FBRA, its content of γ-tocopherol may not be an important contributor to its chemopreventive action. The combination of ferulic acid and γ-tocotrienol has been reported to synergistically inhibit the proliferation of a prostate cancer cell line [30]. In another model, the transgenic adenocarcinoma of mouse prostate (TRAMP) mouse, phytic acid treatment generated a significant reduction in tumor grade and cell proliferation as well as inducing apoptosis in prostate tumor tissues [21]. Furthermore, it has been reported that caffeic acid, phytic acid, and γ-tocotrienol inhibit cancer cell growth in vitro and/or in vivo by activating the AMPK signaling pathway [21,31,32]. Because FBRA inhibits prostate cancer progression in TRAP rats, which lack functional p53 and RB tumor suppressors, the identified chemopreventive action of FBRA is specifically focused on delaying the development of clinically evident disease by suppressing the progression of precancerous lesions to prostate cancer. Recently, we described the establishment of an invasive prostate adenocarcinoma model induced within a shorter period of time by intermittent testosterone propionate administration to TRAP rats [33]. It is possible that the progression of prostate cancer to an invasive phenotype may also be suppressed by FBRA, and this should be assessed in future studies.

## 5. Conclusions

The present investigation using the TRAP model provided evidence that FBRA can suppress prostate carcinogenesis with induction of apoptosis and inhibition of proliferation through enhancing AMPK activation. In consideration of the lack of any toxic changes in organs such as the liver and kidneys, FBRA would appear to be an effective agent for cancer chemoprevention in the prostate.

## Figures and Tables

**Table 1:** The final body weight and the weights of the liver, kidneys, and ventral prostate.

Treatment (No. of Rats)	Body Weight (g)	Liver Weight (g)	Kidney Weight (g)	Ventral Prostate Weight (g)
Control (12)	679.3 ± 47.6	20.2 ± 2.1	3.3 ± 0.3	0.45 ± 0.40
5% FBRA (12)	699.1 ± 45.6	21.8 ± 1.6	3.6 ± 0.3 *	0.34 ± 0.06
10% FBRA (12)	703.4 ± 63.3	21.2 ± 1.7	3.5 ± 0.3	0.38 ± 0.09

Data are means ± standard deviation. * *p* < 0.05 compared with control group.

**Table 2:** Incidence of carcinoma and histological evaluation of neoplastic lesions in the prostate of TRAP rats treated with FBRA.

Treatment (No. of Rats)	Ventral Prostate				Lateral Prostate			
	Incidence of Carcinoma (%)	% of Lesions in Prostate			Incidence of Carcinoma (%)	% of Lesions in Prostate		
		LG-PIN	HG-PIN	Adenoca.		LG-PIN	HG-PIN	Adenoca.
Control (12)	12 (100)	8.6 ± 3.5	75.7 ± 8.1	15.7 ± 9.1	12 (100)	13.6 ± 9.0	77.0 ± 9.4	9.4 ± 5.0
5% FBRA (12)	12 (100)	8.6 ± 3.2	79.1 ± 3.2	12.3 ± 4.1	12 (100)	12.2 ± 4.7	80.7 ± 4.6	7.1 ± 3.1
10% FBRA (12)	12 (100)	12.9 ± 3.9 **	75.0 ± 7.6	12.1 ± 7.1	9 (75)	21.4 ± 14.8	74.2 ± 14.1	4.4 ± 5.3 *

Data are means ± standard deviation, * *p* < 0.05, ** *p* < 0.01 compared with control group (1-way analysis of variance and Dunnett’s post-hoc test).

## Abbreviations

The following abbreviations are used in this manuscript:

VP	ventral prostate
LP	lateral prostate
LG-PIN	low grade intraepithelial neoplasia
HG-PIN	high grade intraepithelial neoplasia
AMPK	AMP-activated protein kinase
FASN	fatty acid synthase
